# Use of Transversus Abdominis Plane (TAP) Blocks for Postoperative Pain Management in a Patient With an Open Abdomen: A Case Report and Review of Literature

**DOI:** 10.7759/cureus.12739

**Published:** 2021-01-16

**Authors:** Simon Berhe, Fabian Kraus, Mohammed Tariq Hanifi, Kamen Vlassakov, Matthias Stopfkuchen-Evans

**Affiliations:** 1 Department of Surgery, Columbia University Irving Medical Center, New York, USA; 2 Department of Obstetrics and Gynecology, Klinikum der Universität München, München, DEU; 3 Department of Anesthesiology, Perioperative and Pain Medicine, Brigham and Women’s Hospital, Boston, USA

**Keywords:** transversus abdominis plane, open abdomen, multi-modality pain management

## Abstract

In light of the superior analgesia and opioid sparing effects provided by transversus abdominis plane (TAP) blocks, numerous new techniques and applications have evolved. However, TAP blocks are still underutilized in the critical care setting, and PubMed‑listed reports on the relevance of TAP integrity for TAP block efficacy are lacking. Here, we report bilateral TAP blocks delivering quick, potent and durable pain relief to a patient with open abdomen (OA) after prior management with opioids and epidural anesthesia had failed. Extending TAP block application to OA patients even in the post‑operative setting might hence reduce opioid consumption and quicken reconvalescence.

## Introduction

Transversus abdominis plane (TAP) blocks are increasingly used as an effective and safe component of multimodal pain management concepts following both laparoscopy or laparotomy [1]. As a result, a range of distinct landmark based and ultrasound-guided methods have emerged to provide postoperative analgesia to the anterolateral abdominal wall [2]. In most cases, TAP blocks are applied under general anesthesia, either pre- or postoperatively. Yet, despite increasing empirical evidence, published data analyzing the essentials of TAP block efficacy remain inconclusive [3]. In particular, reports on the significance of TAP integrity for TAP block efficacy are lacking.

In this case, we describe the application of bilateral TAP blocks at bedside in a patient with open abdomen (OA) status post emergency laparotomy after prior attempts to deliver sufficient pain control with opioids and epidural anesthesia had failed. According to our PubMed search, this is the first time in the last 20 years a TAP block has been described as delivering rapid, potent and durable postoperative pain relief in an OA patient.

## Case presentation

A 71-year-old, multimorbid male patient presented with right-sided abdominal pain to the emergency department; a CT scan of his abdomen was significant for superior mesenteric artery (SMA) stenosis, severe stenosis of the inferior mesenteric artery (IMA), pneumatosis coli of the ascending colon, portal venous gas, as well as a 50% bilateral stenosis of the renal arteries.

The patient was therefore taken immediately to the operating room for exploratory laparotomy with resection of the necrotic bowel, a right-sided hemicolectomy with cecectomy. At the end of the operation, it was decided to leave the abdomen open given the lack of Doppler vascular signal in the transverse colon, which otherwise appeared macroscopically viable. The patient was taken to the intensive care unit (ICU) and postoperatively intubated for his OA with eventual return to the operating room for definitive closure.

Postoperative pain was initially managed by a continuous epidural catheter infusion of bupivacaine 0.125% at 6 ml/h, as well as by intravenous fentanyl infusion. However, pain control still remained insufficient, such that concerns arose as to whether the epidural catheter was dislocated. It was consequently removed and a second epidural was performed at the thoracic level (T8-9) in a standard fashion without complication. Yet, even with a bolus of 3 ml of 2% lidocaine through the epidural, the patient's pain remained intolerable. At this point, he extubated himself, which we classified rather as a sign of pain excess than of post-operative delirium, as the patient appeared to be orientated during his entire course and expressed clearly that his pain level was 9 out of 10 on a visual rating scale. 

Increasing the patient’s analgesic doses did not seem like a feasible option given his advanced age and the already exhibited signs of somnolence and respiratory depression from escalating doses of his continuous fentanyl infusions. Another neuraxial approach seemed promising, but the patient was started on therapeutic anticoagulation for his critical SMA stenosis and severe stenosis of the proximal IMA. Against this background, it was decided to proceed with a TAP block. 

**Figure 1:**
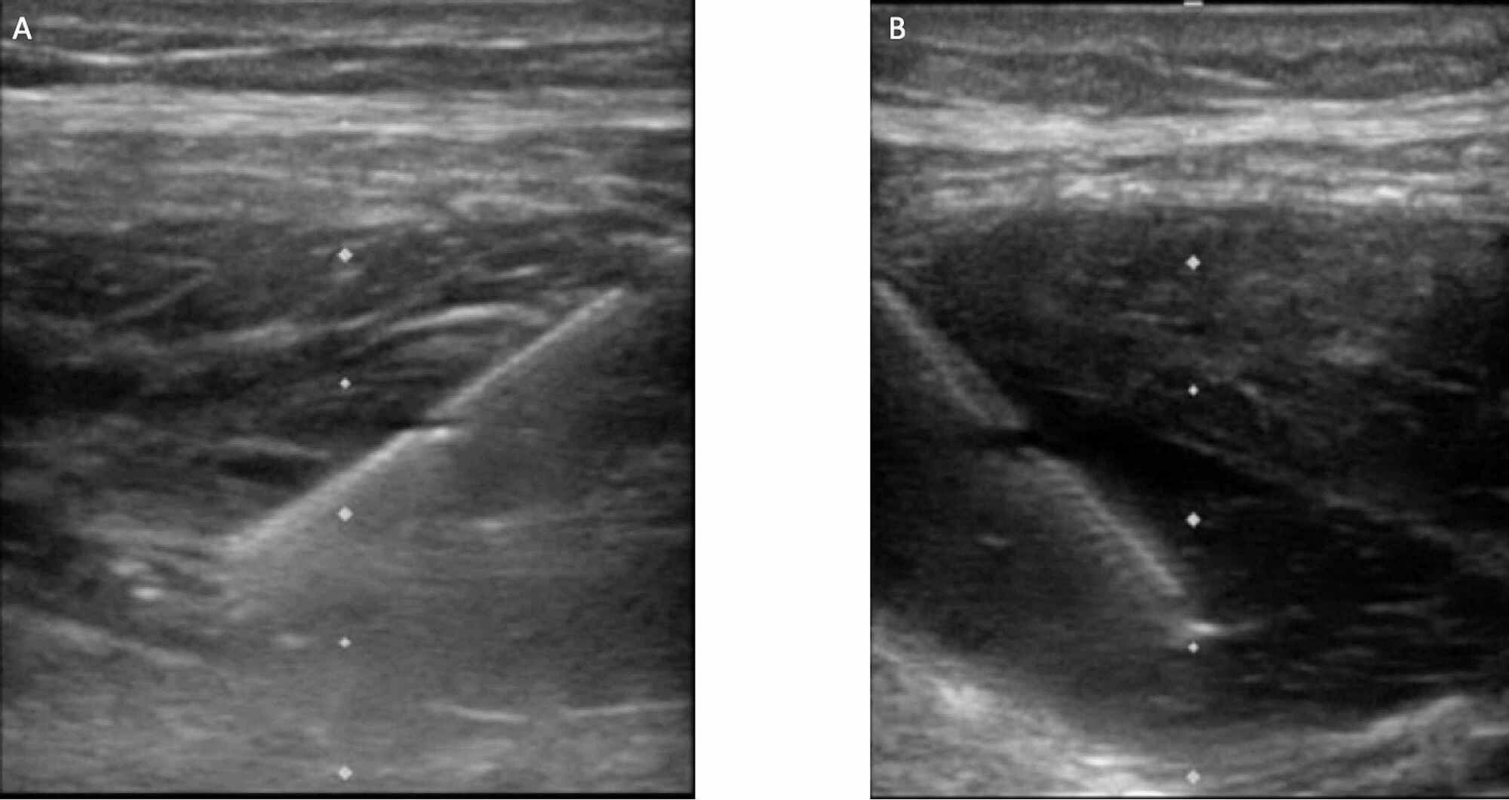
Ultrasound guided deposition of local anesthetic in the right (A) and left (B) transversus abdominal plane (TAP) to block somatic sensibility of the anterolateral abdominal wall (T7 to L1)

A 5 cm, 21 g x 100 mm Pajunk Sonoplex Stim Cannula (Pajunk, Geisingen, Germany) needle was applied in standard fashion and under ultrasound guidance using a Sonosite US M-Turbo (SonoSite Inc., Bothell, Washington, USA) with an L25 Linear Probe for visualization of fascial plane sonoanatomy (Figure 1). In total, a comparatively low concentration of 60 ml of 0.25% Marcaine with epinephrine 1:200,000 was injected in two equally divided doses bilaterally to each TAP. The patient tolerated the procedure well. 

**Figure 2:**
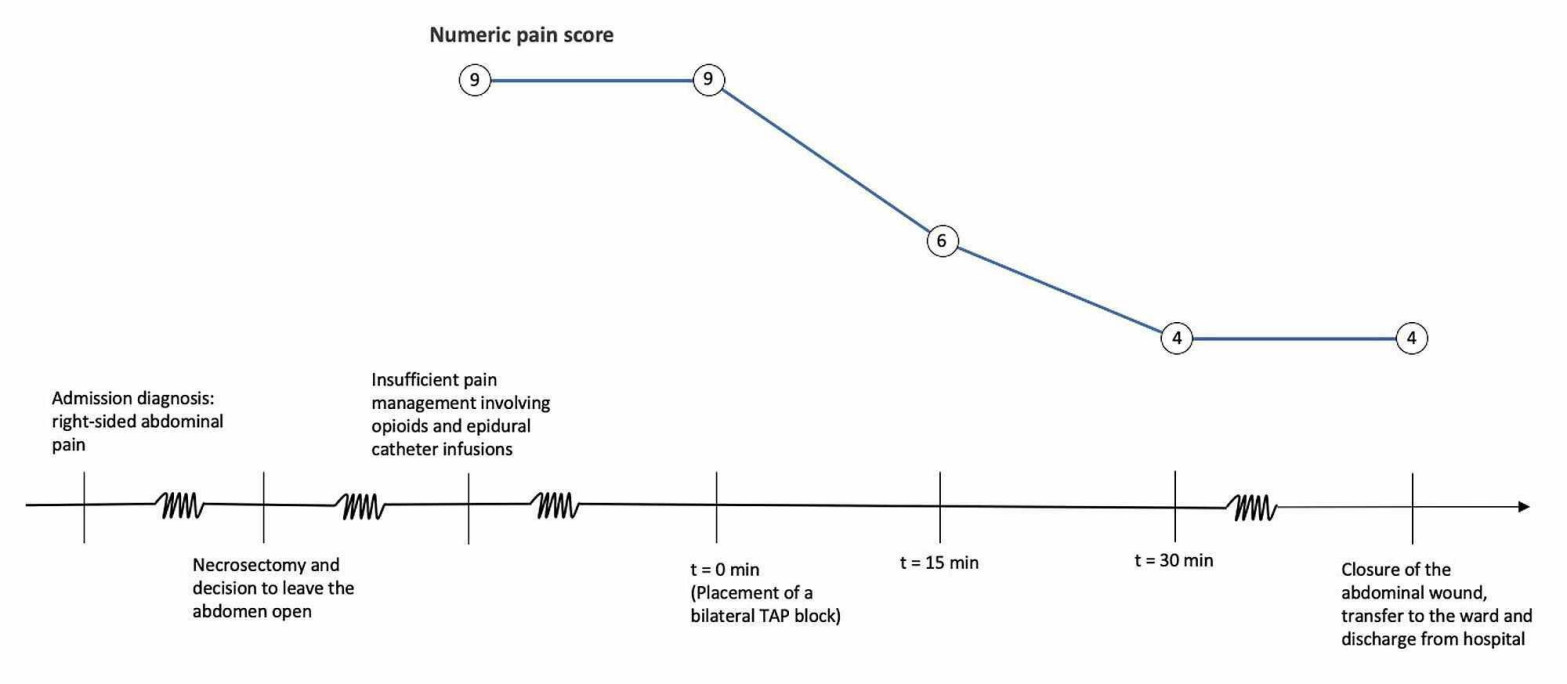
Timeline of the patient’s treatment course TAP: transversus abdominis plane.

His pain was assessed after 15 minutes, 30 minutes, and one hour on the visual rating scale. Fifteen minutes after injection, his pain had improved from 9 to 6 out of 10, and further dropped to 4 after 15 minutes. The following day, he was brought to the operating room for abdominal closure. The remainder of his hospital stay was unremarkable, without any episodes of pain excess under gradually decreasing opioid consumption (Figure 2).

## Discussion

TAP blocks have emerged as a potent tool for immediate postoperative pain management following laparoscopic or open surgery by injection of local anesthetics (LA) in the anatomical space between the internal oblique and transversus abdominis muscles (TAP), where the anterior rami of the lower six thoracic nerves (T7-T12) as well as of the first lumbar nerve (L1) penetrate the plane and arborize, providing somatic sensibility to the anterolateral abdominal wall [4,5]. However, the consensus amongst experts on volume, dosage, and even the most suitable analgesic agent itself has not yet been reached, with ropivacaine and bupivacaine arguably ranging among the most frequently used LA in TAP blocks to date [6].

Equally, the precise method of TAP block delivery varies widely. After initial reports on landmark based techniques, ultrasound-guided methods began to evolve [2]. To date, anesthesiologists have chosen from a variety of TAP block techniques, such as the posterior, lateral, or oblique subcostal approach. 

The posterior technique consists of an injection in the lumbar triangle of Petit, which is bordered caudally by the iliac crest, posteriorly by the latissimus dorsi muscle, and anteriorly by the external oblique muscle [7]. It results in reliable anesthesia of the anterolateral, supra-, and infraumbilical abdominal wall [8]. The lateral approach requires ultrasound-guided deposition of LA at the midaxillary line between costal margin and iliac crest, whereas for the oblique subcostal technique anesthetics are injected between xiphoid and the anterior part of the iliac crest [8,9]. In comparison to the posterior technique, Lissauer et al. report a tendency towards reduced dermatome coverage with the lateral approach and a better coverage of T6-T9 dermatomes with the oblique subcostal approach [10]. Differences in efficacy of the three TAP block techniques might derive from the wide spectrum of variables, e.g. type, volume, and dose of LA, the exact point in time (pre- or postsurgical) as well as the applied technique itself [11].

Additional variations of the presented approaches for TAP blocks can be achieved by multi-injection or catheter-based continuous infusion techniques, or by performing intra-operative blocks under direct vision instead of ultrasound-guidance [12].

Although the analgesic potential of TAP blocks is constantly being refined, it has already proven itself as a potent option in multimodal post-operative pain management. In a prospective double-blinded, randomized controlled trial, Bharti et al. observed significant reduction in morphine consumption, lower pain scores, and reduced incidence of sedation in the TAP group after laparotomy [13]. In addition, McDonnell et al. showed considerable improvement of reduced visual analog scale pain scores, and also marked reduction of the use of morphine in patients with TAP blocks after abdominal surgery [14].

Hence, TAP blocks might bear the potential to supplement or even surrogate at least two, up to now still important, elements of modern multimodal pain management concepts: opioids and epidural analgesia.

Since most frequent opioid side effects are dose-dependent, the reduction of opioid consumption decreases the likelihood of postoperative nausea and vomiting, pruritus, urinary retention, ileus, sedation, and respiratory depression [11,15]. In addition, patients regain gastrointestinal function more quickly and can be discharged from the hospital earlier [11]. Nevertheless, intrathecal morphine administration seems to be more potent than TAP blocks, according to Brogi et al. Further investigations must be carried out to compare the pain management via TAP block with local infiltration techniques as well as with ilioinguinal-iliohypogastric nerve blocks [16].

Conventional epidural anesthesia is resource-consuming, limits patient mobility, and bears the risk of negatively affecting the cardiovascular and gastrointestinal tract. Nevertheless, it is an elaborate means of reducing opioid consumption. It has been shown that acute post-operative pain management via peridural anesthesia improves patient outcome far more than via opioids in terms of a better post-operative bowel function, a reduction in the incidence of post-operative myocardial infarction or pneumonia, and when weaning patients off artificial respiration [17,18]. 

It is precisely because of their positive impact on pain reduction, morphine consumption, and patient mobility that TAP blocks might come into play as an ideal alternative to epidural anesthesia, compared to which they carry a lower risk of vasoparesis, hypotension, splanchnic hypoperfusion, and prolonged and unwanted motor blockade [19]. 

Moreover, TAP blocks can also be carried out when an epidural analgesia is contraindicated, e.g. in coagulopathic or septic conditions, and might even reduce chronic post-operative pain by preventing pain memory formation in more deeply sedated patients [17,20].

## Conclusions

Despite the increasing use of TAP blocks and encouraging results, the essentials of TAP block efficacy still remain under investigation. As an example, the question of whether TAP blocks can also provide pain relief in patients whose abdomen has had to be kept open, and who were hence left with a disrupted TAP integrity, had thus far remained unanswered. In this case report, we present a patient in strong need of pain relief after prior attempts to control pain with opioids and epidural anesthesia had failed. Increasing the opioid dose was not possible given the patient’s advanced age and beginning signs of somnolence in addition to respiratory depression. The anticoagulation therapy that the patient received for his critical SMA and IMA stenosis precluded a second neuraxial approach. Although our PubMed search for the time between June 2000 and December 2019 did not provide any clarification as to its applicability to patients with OA, a TAP block was employed in this case. The most prevalent concern was that LA would not be sufficiently conserved nor evenly distributed within the TAP when the abdominal anatomy was abrogated, limiting postsurgical applicability of TAP blocks on patients with a closed abdominal midline incision. However, 15 minutes after placement of the TAP block, the pain level had already improved from 9 out 10 on the numeric rating scale to 6, and to 4 after a subsequent 15 minutes. The pain level remained stable and opioid consumption could gradually be reduced until the abdomen was closed on the following day. As, in this case, only single shots of submaximal doses of the LA were applied, even greater efficacy might be achieved by escalating the dose of LA and using catheter-based injection methods. Moreover, additional research to identify the LA with the strongest effects might increase the potency of this method even further. In conclusion, we endorse the expanded application of TAP blocks in the critical care setting and particularly to OA patients, where they can improve pain control, reduce opioid consumption, increase patient mobility and ultimately speed up patient reconvalescence.
